# Supplementary material for: PAUF Induces Migration of Human Pancreatic Cancer Cells Exclusively via the TLR4/MyD88/NF-κB Signaling Pathway
Source: Int J Mol Sci. 2022 Sep 27;23(19):11414. doi: 10.3390/ijms231911414 (PMC9570212; doi:10.3390/ijms231911414)
Supplement: Supplementary file 1 [file ijms-23-11414-s001.zip › ijms-1897822-supplementary.pdf]

A

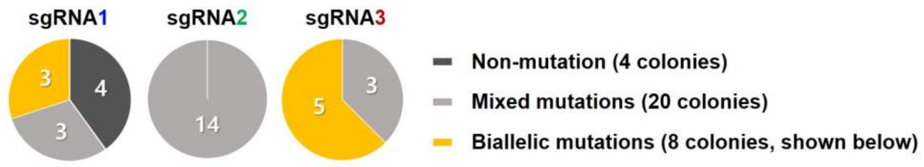

B

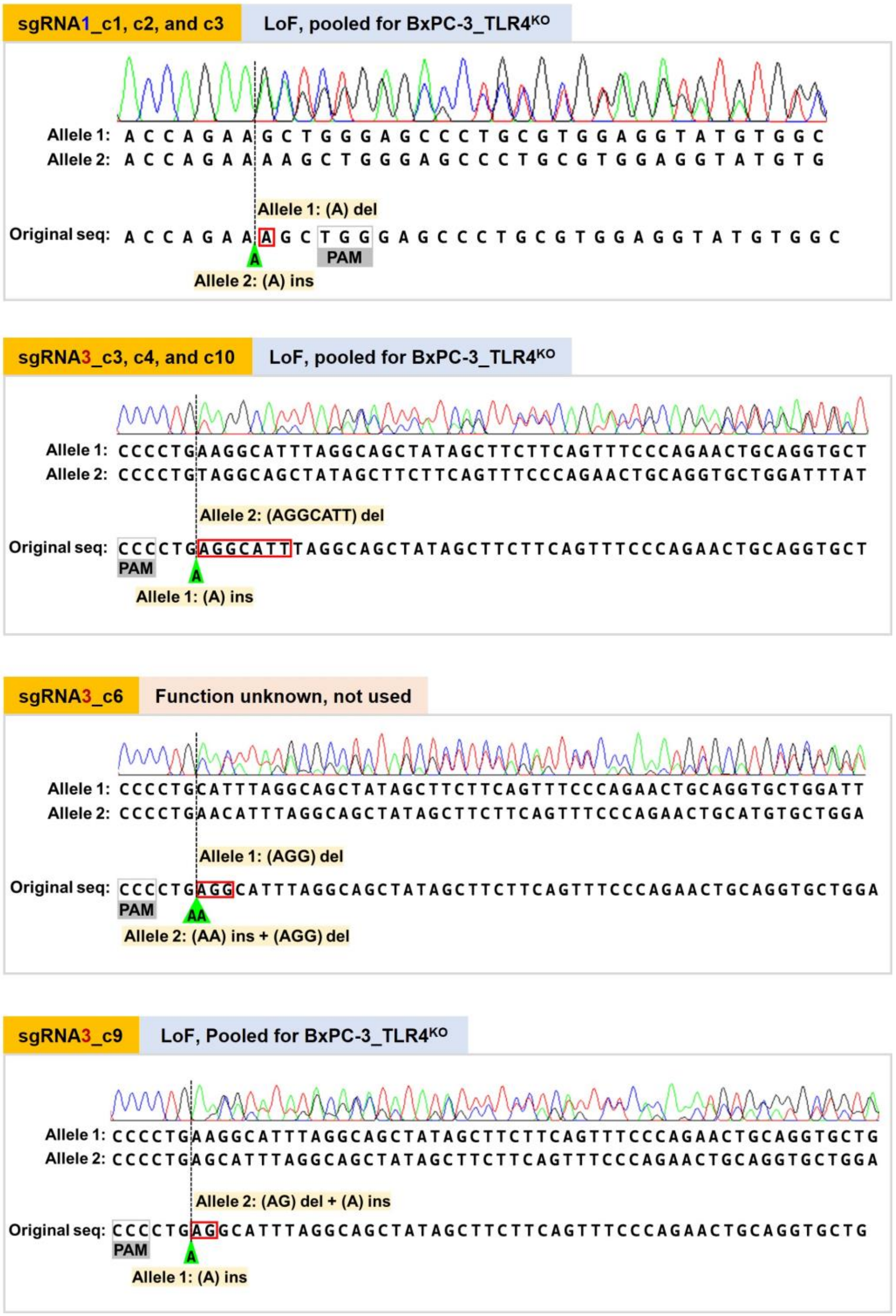

Supplementary Figure S1. Eight single clones with biallelic mutations in the TLR4 gene were obtained. (**A**) A total of thirty-two single colonies were picked after BxPC-3 cells were transfected with TLR4 sgRNA1, sgRNA2, or sgRNA3, which have either non-mutation, or mixed mutations, or biallelic mutations. (**B**) Sanger sequencing results of eight single clones with biallelic mutations. Dash line indicates 3 bp upstream of protospacer adjacent motif (PAM) site. LoF = loss-of-function; del = deletion; ins = insertion.

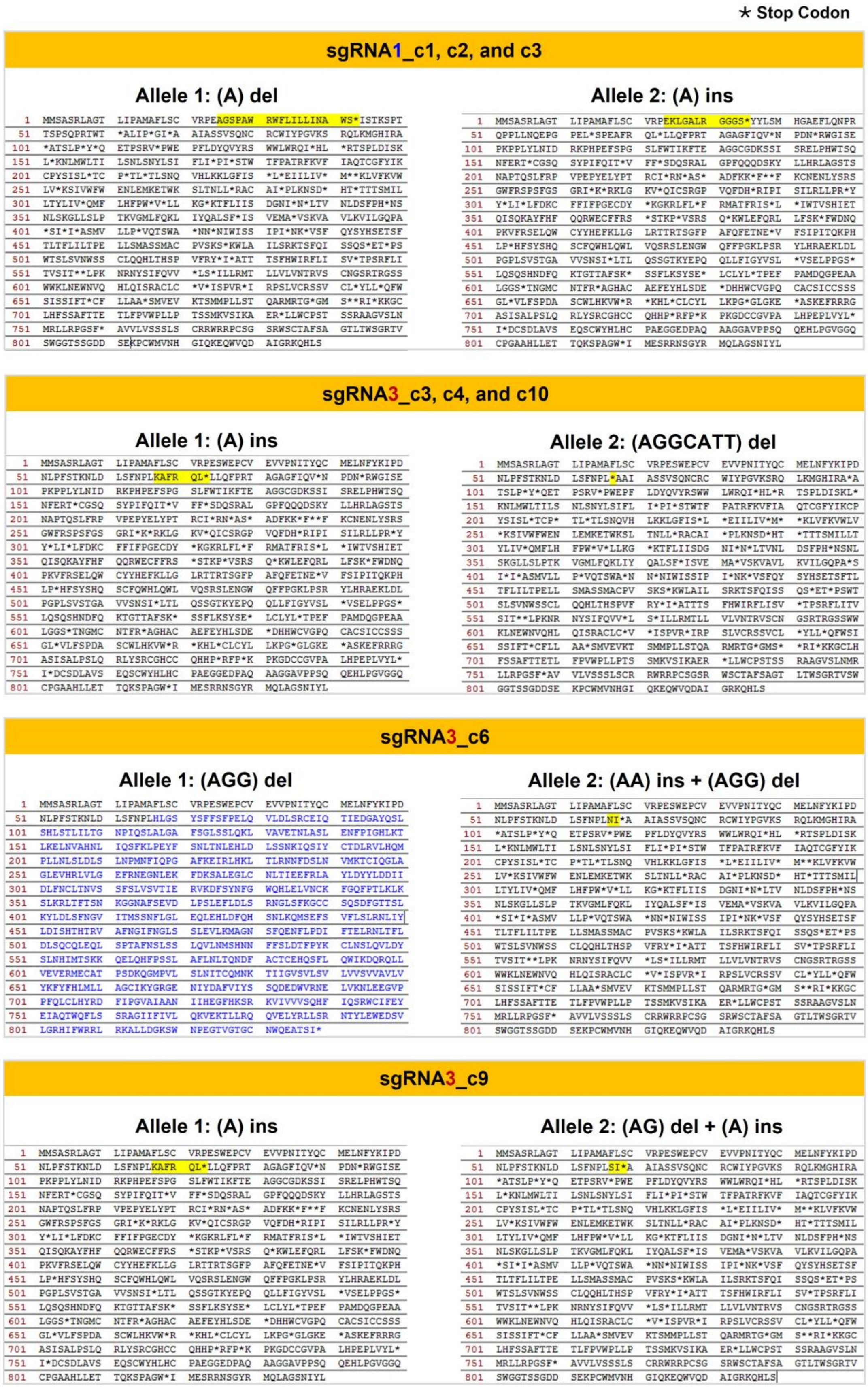

Supplementary Figure S2. Prediction of amino acid sequences of the eight single clones with biallelic mutations in the TLR4 gene using Vector NTI software. Highlighted parts show stop codon (denoted by asterisk *) caused by the mutations. In sgRNA3_c6, the AGG deletion in allele 1 resulted in one amino acid deletion of TLR4 rather than premature translation termination. del = deletion; ins = insertion.

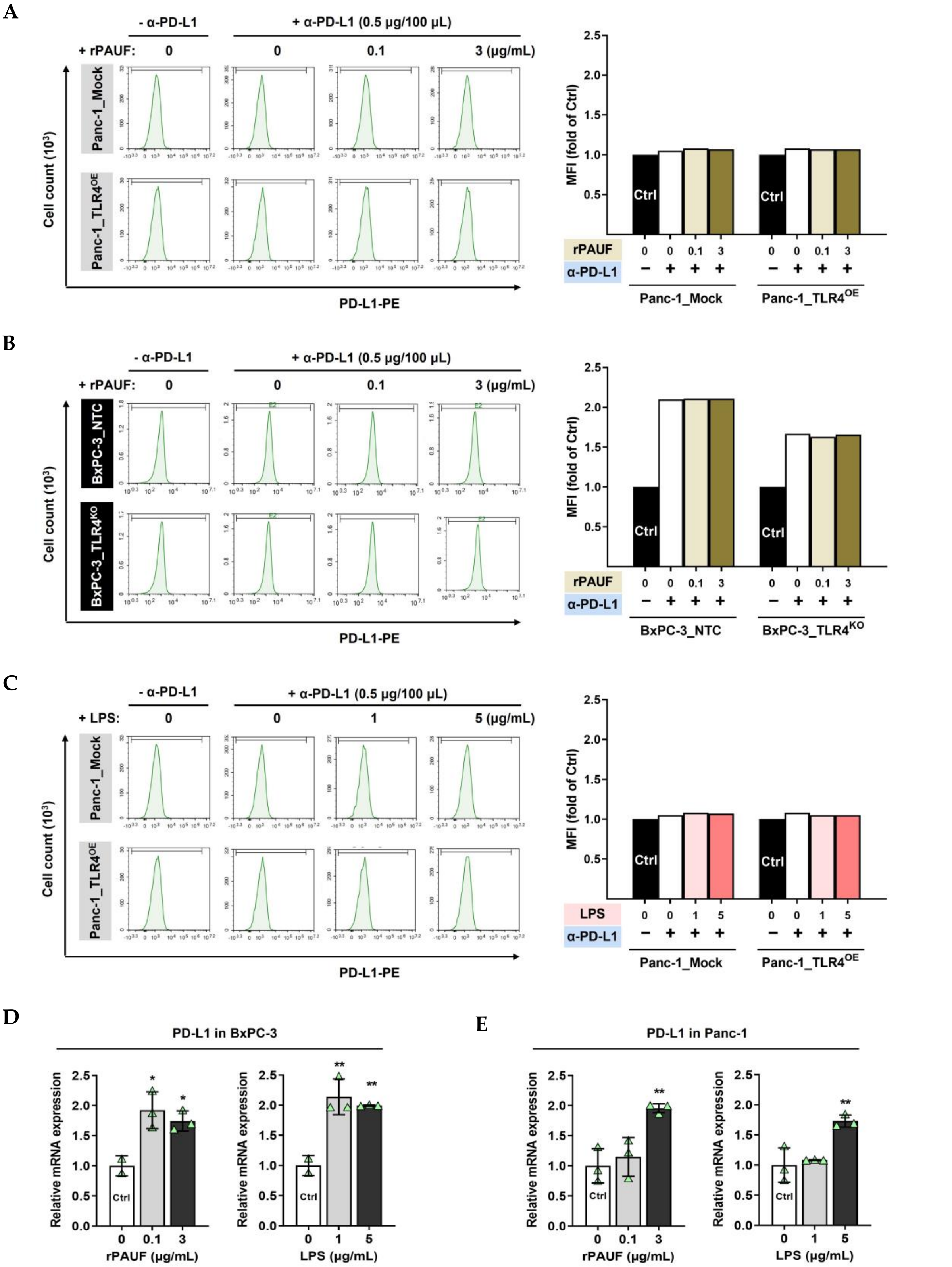

Supplementary Figure S3. rPAUF does not impact cell surface PD-L1 expression, but increases PD-L1 expression in cytoplasm, of pancreatic cancer cells. And PD-L1 expression in cells with higher basal surface PD-L1 expression (BxPC-3>>Panc-1), is more sensitive to TLR4 activity change. Flow cytometry showed cell surface PD-L1 expression in (**A**) Panc-1_Mock and Panc-1_TLR4OE cells, and in (**B**) BxPC-3_NTC and BxPC-3_TLR4KO cells, after the cells were treated by 0, 0.1, or 3 μg/mL of rPAUF for 24 hr, and in (**C**) Panc-1_Mock and Panc-1_TLR4OE cells after cells were treated by 0, 1, or 5 μg/mL of LPS for 24 hr. mRNA expression of PD-L1 in (**D**) BxPC-3 and (**E**) Panc-1 cells, after the cells were treated by rPAUF (0, 0.1, or 3 μg/mL) or LPS (0, 1, or 5 μg/mL) for 6 hr.
